# Recombinant B87 and HLJ0504 VP2 Vaccines Protect Chickens Against Infectious Bursal Disease

**DOI:** 10.3390/vetsci13101033

**Published:** 2026-09-29

**Authors:** Tayyab Ali Asif, Pengyu Chen, Jiahao Li, Jing Zhang, Pengchao Ji, Dawei Jiang

**Affiliations:** 1 International Joint Research Center of National Animal Immunology, College of Veterinary Medicine, Henan Agricultural University, Zhengzhou 450046, China; tayyab.nibge@gmail.com (T.A.A.); chen2780273669@163.com (P.C.); 13949046414@163.com (J.L.); m18737642209@163.com (J.Z.); 2 Longhu Laboratory, Zhengzhou 450046, China

**Keywords:** IBDV, virus-like particles, humoral immune response, *Escherichia coli* expression, vaccine safety, protective immunity, bursa of Fabricius

## Abstract

Infectious bursal disease virus (IBDV) causes immunosuppression and bursal damage in chickens, and safer recombinant vaccines that provide durable protection are needed. This study compared the antigenicity, immunogenicity, protective efficacy, safety and antibody persistence of unpurified VP2 preparations and purified VP2 virus-like particles (VLPs) derived from the B87 and HLJ0504 IBDV strains. Recombinant VP2 proteins were expressed in *Escherichia coli*, purified and characterized, formulated with oil adjuvant and evaluated in specific-pathogen-free White Leghorn chickens. Both VP2 proteins retained antigenicity and assembled into relatively uniform VLPs of approximately 25 nm. Purified VLPs induced stronger antibody responses than corresponding unpurified preparations, with 10 µg producing the highest responses. In a virus neutralization assay, HLJ0504 VP2-based preparations induced the highest neutralizing-antibody titers. Following challenge with virulent IBDV BC6/85, 10 µg of HLJ0504 VLPs and unpurified HLJ0504 VP2 at an agar-gel precipitation titer of 1:10 each protected nine of 10 chickens, similar to a commercial inactivated vaccine, and preserved bursal architecture. No systemic or injection-site adverse reactions were detected and selected recombinant vaccines maintained elevated serum-antibody levels for 20 weeks. Thus, HLJ0504 VP2-based vaccines, particularly the VLPs formulation, are promising safe and protective candidates for controlling infectious bursal disease.

## Simple Summary

Infectious bursal disease is a contagious viral illness of chickens that damages a bursa of Fabricius needed for normal disease resistance, leading to weakened immunity and major losses for poultry farmers. Existing vaccines do not always protect this organ equally well or maintain strong protection for long periods. This study aimed to develop safer, non-infectious vaccines using the main outer protein of the virus from two virus strains, B87 and HLJ0504, produced in a bacterial production system. We compared less-processed protein preparations with purified proteins that formed tiny virus-shaped particles unable to cause disease. In vaccinated chickens, the purified particles produced stronger antibody responses, especially at higher doses, and the HLJ0504 particle vaccine produced the strongest virus-blocking antibodies. After chickens were exposed to a harmful virus, the best HLJ0504 vaccines protected nine out of ten birds, matching the protection of a commercial inactivated vaccine, and helped keep the immune organ structurally normal. The vaccines caused no obvious side effects, were absorbed well at the recommended dose, and maintained antibody levels for twenty weeks. These findings suggest that this vaccine approach could provide a safe, practical way to control infectious bursal disease and improve poultry health and food security.

## 1. Introduction

IBDV causes acute damage to the bursa of Fabricius, the central B-cell lymphoid organ in chickens [1]. Virulent IBDV infection may induce bursal swelling or atrophy, hemorrhage, gelatinous exudation, lymphoid-cell necrosis, inflammatory infiltration, and disruption of follicular architecture [2]. Effective vaccination must therefore prevent both clinical manifestations such as depression, anorexia, watery diarrhea, soiled vents, prostration, and incoordination, and structural bursal injury, an important indicator of protective efficacy [3].

Commercial live attenuated, inactivated, and subunit vaccines are used to control infectious bursal disease [4]. However, these formulations differ in the onset, magnitude, and persistence of antibody responses and in their ability to protect bursal tissue after virulent-virus challenge [4]. These differences highlight the need for recombinant antigens that combine protective immunogenicity with safety, production consistency, and practical administration.

The major capsid protein VP2 is a promising vaccine antigen because it can be produced without infectious virus and assembled into particulate structures [5]. *Escherichia coli* provides a practical system for VP2 production without propagating live IBDV [6]. Recombinant VP2 may be used as a soluble antigen in crude bacterial preparations or purified and assembled into VLPs. Because antigen purity and physical form can influence vaccine performance [7], direct comparison of these formats is essential. Unpurified VP2 offers a simple, economical production strategy, whereas purified VLPs provide a more defined particulate antigen. Their relative immunogenicity and protective efficacy may vary with viral strain, dose, formulation, and administration conditions [8]. The selection of B87 and HLJ0504 was intended to compare VP2 antigens derived from biologically distinct IBDV backgrounds. B87 is an established vaccine-associated strain, whereas HLJ0504 represents a very virulent IBDV (vvIBDV) lineage identified in China [9,10]. VP2 is the principal capsid antigen of IBDV and contains major conformational neutralizing epitopes, particularly within its hypervariable region; sequence variation in this region can alter antigenic properties and antibody recognition [11]. Thus, comparing VP2 derived from B87 and HLJ0504 provides an opportunity to determine whether strain origin is associated with differences in recombinant-antigen characteristics and vaccine-associated immune responses. Previous studies have demonstrated the potential of recombinant VP2 and VP2-based particles as IBDV vaccine candidates, including approaches incorporating VP2 from very virulent strains [11]. However, direct comparative evaluation of B87- and HLJ0504-derived VP2 in both unpurified and purified VLPs formats under the same experimental framework remains limited. The present study therefore compared these two VP2 sources across antigenicity, VLPs assembly, humoral and virus-neutralizing-antibody responses, protection against virulent IBDV challenge, local safety and absorption, and persistence of serum-antibody responses. Recombinant VP2 preparations can be quantified by agar-gel precipitation or protein-concentration assays and formulated with oil adjuvant at different antigen levels [9,10]. Immunogenicity can be assessed through serum IBDV-specific antibodies, whereas protection can be evaluated by virulent-virus challenge and gross and histopathological examination of the bursa [12]. Safety, local absorption, administration volume, and antibody persistence are also important selection criteria [13].

Their immunogenicity in specific-pathogen-free chickens was compared with commercial subunit, inactivated, and attenuated vaccines. Neutralizing-antibody responses induced by HLJ0504 VLPs and B87 VLPs were further evaluated using a DF-1 cell-based virus neutralization assay. Protective efficacy was determined by challenge with virulent IBDV BC6/85, followed by gross and histopathological bursal evaluation. Vaccine safety, local absorption at different administration volumes, and serum-antibody persistence for up to 20 weeks were assessed to identify a safe, immunogenic, and protective recombinant VP2 formulation for further development as an IBDV subunit vaccine.

## 2. Materials and Methods

### 2.1. Recombinant Strains, Viruses, Animals and Ethics

Recombinant Escherichia coli strains carrying codon-optimized VP2 genes from IBDV strains B87 (GenBank DQ202329.1) and HLJ0504 (GenBank GQ451330.1) were maintained at the National Joint Research Center for Animal Immunology. The virulent IBDV BC6/85 strain, used for challenge, was obtained from the China Institute of Veterinary Drug Control.

Specific-pathogen-free White Leghorn chickens were obtained from Beijing Merial Vital Laboratory Animal Technology Co., Ltd. (Beijing, China) and maintained in negative-pressure, filtered-air isolation units. All procedures were approved by the Animal Ethics Committee of the Henan Academy of Agricultural Sciences (LLSC41022054). Chickens were euthanized by cervical dislocation under anesthesia with xanthazine when required.

### 2.2. VP2 Expression and Purification

Codon-optimized B87 and HLJ0504 VP2 genes were inserted separately into pET-28a through the *Bam*HI and *Xho*I sites, generating pET28a-optiVP2 constructs. Recombinant plasmids were transformed into *E. coli* BL21 (DE3) pLysS (Novagen, Beijing, China) and verified by PCR and restriction digestion.

Recombinant bacteria were cultured in Luria-Bertani (LB) broth [14] containing 100 µg mL^−1^ kanamycin at 37 °C to an OD_600_ of 0.5–0.7. B87 and HLJ0504 VP2 expressions were induced with 0.2 and 0.5 mM isopropyl β-D-1-thiogalactopyranoside (IPTG), respectively, for 21 h at 37 °C. Cells were harvested, disrupted by sonication and centrifuged at 9000 rpm for 15 min at 4 °C [15].

Expressed *E.coli* VP2-containing cells were lysed through sonication (05 s pulse on, 05 s pulse off, 40% amplitude, and 15 cycles), and centrifuge at 5000× *g* for 10–15 min at 4 °C to collect lysate. VP2 proteins were purified from clarified lysates by Ni-NTA affinity chromatography [15], followed by concentration through 20% sucrose and separation on a discontinuous 20%, 40% and 60% sucrose gradient at 40,000 rpm and 4 °C for 10 h [16]. Material collected from the 40–60% interface was resuspended in phosphate-buffered saline (PBS). Protein expression and purity were assessed by sodium dodecyl sulfate–polyacrylamide gel electrophoresis (SDS-PAGE) and Western blotting (WB). Briefly, protein samples were mixed with 4× loading buffer and heated at 99 °C for 10 min. Samples (20 µL per lane) were separated on a 12% SDS-PAGE gel at 120 V for approximately 90 min. For SDS-PAGE analysis, gels were stained with Coomassie Brilliant Blue R-250 [16]. For Western blotting, proteins were transferred onto a PVDF membrane at 100 V for 60 min at 4 °C. The membrane was blocked with 5% skim milk in TBST at room temperature for 1 h, then incubated with a mouse anti-His monoclonal antibody (1:3000 dilution) at 4 °C overnight, followed by HRP-conjugated goat anti-mouse IgG secondary antibody (1:5000 dilution) at room temperature for 1 h. Protein bands were visualized using an ECL substrate. Protein concentrations were determined using a bicinchoninic acid (BCA) assay with a commercially available kit (Pierce™ BCA Protein Assay Kit, Thermo Fisher Scientific, Waltham, MA, USA). Briefly, 25 µL of each protein sample or bovine serum albumin (BSA) standard was mixed with 200 µL of BCA working reagent (Reagent A: Reagent B, 50:1, *v*/*v*) in a 96-well microplate. The plate was incubated at 37 °C for 30 min, and the absorbance was measured at 562 nm using a microplate reader. Protein concentrations were calculated from the BSA standard curve (R^2^ > 0.99). All samples were measured in triplicate [17].

### 2.3. VLPs Characterization and Agar-Gel Precipitation Assay

Purified VP2 proteins were suspended in PBS to permit VLPs assembly. Samples were adsorbed onto carbon-supported 200-mesh nickel grids, negatively stained with 3% phosphotungstic acid at pH 6.8 and examined using a JEM-1400 transmission electron microscope (TEM) (Tokyo, Japan) [18]. Particle size distributions were additionally determined by dynamic light scattering (DLS) [19].

The agar-gel precipitation (AGP) assay was used to assess soluble VP2 antigens in bacterial lysates, evaluate purified VLPs preparations and detect IBDV antigens after challenge. Serial twofold antigen-dilutions were placed around a central well containing reference IBDV-positive antiserum and incubated at 37 °C for 72 h. Samples were considered positive when their precipitation lines merged with the reference line. The AGP titer was defined as the highest dilution producing a visible precipitation line.

### 2.4. Vaccine Preparation and Immunization

Unpurified B87 and HLJ0504 VP2 preparations were adjusted to AGP titers of 1:10, 1:20 or 1:40. Purified VLPs preparations were adjusted to 4, 20 or 40 µg mL^−1^, corresponding to doses of 1, 5 or 10 µg per chicken. Antigens were emulsified with white-oil adjuvant 7# at an aqueous antigen-to-oil ratio of 1:2. PBS emulsified with the same adjuvant served as the adjuvant control. The unpurified VP2 preparations were standardized by AGP titers, whereas the purified VLPs were dosed by total protein mass.

Specific-pathogen-free (SPF) chickens were allocated to predefined experimental groups such as unpurified VP2, purified VP2 VLPs and control groups according to the study design in Table 1. Each treatment group contained 10 chickens, except for the unchallenged blank-control group, which contained five birds. At 28 days of age, experimental groups received 250 µL of the assigned formulation intramuscularly. Commercial subunit vaccine (Qingdao Yebio Biological Engineering Co., Ltd., Qingdao, China), inactivated vaccine water-in-oil emulsion (Huahong biology, Meizhou, China) and intermediate-virulence attenuated B87 vaccines (Guangxi Liyuan Biological Co., Ltd., Guangxi, China) were administered according to the manufacturers’ instructions. At challenge, PBS and unchallenged controls were included. The complete experimental design is presented in Table S1 (File S1). Blood was collected from the brachial vein at two-week intervals, and sera were separated for antibody analysis. 

### 2.5. Virulent-Virus Challenge and Assessment of Protection

Because BC6/85 was replicated in chicken embryo fibroblasts, challenge-virus stocks were propagated in susceptible chickens. Four-week-old SPF chickens were inoculated orally, and bursae of Fabricius were collected at 3 days post-infection, homogenized in PBS at 1:10 and stored at −80 °C.

The 50% bursa infective dose (BID_50_) was calculated using the Kärber method: logBID_50_ = L − d (S − 0.5), where (L) is the logarithm of the lowest dilution, (d) is the interval between consecutive logarithmic dilutions and (S) is the sum of the proportions of chickens showing bursal lesions [20].

At 28 days after vaccination, all groups except the unchallenged control were orally challenged with virulent BC6/85 at 10^−5^ BID_50_ per bird. Clinical signs and survival were monitored for 4 days. Chickens were then euthanized, and the muscles, bursa of Fabricius, liver, kidneys and other major organs were examined. The bursa-to-body-weight ratio was calculated as bursal weight/body weight × 1000. Following virulent IBDV challenge, individual protection status was determined based on gross pathological examination of the BF. Chickens showing bursal swelling or atrophy, hemorrhage, yellow discoloration or gelatinous exudation were classified as unprotected; those without these lesions were classified as protected.

### 2.6. Serology, Virus Neutralization Test, and Histopathology

Serum IBDV-specific antibodies were measured using a commercial enzyme-linked immunosorbent assay (ELISA) kit (IDEXX, Elmhurst, IL, USA) according to the manufacturer’s instructions, with absorbance recorded at 650 nm.

Bursae collected after challenge were fixed in formalin, routinely processed, sectioned and stained with hematoxylin and eosin (H&E) [1]. Gross pathological evaluation and histopathological scoring were performed by investigators blinded to treatment allocation. Lesions were scored from 1 to 4 according to predefined criteria. Score 1, normal follicles or mild lymphocyte depletion; score 2, moderate lymphoid damage with focal necrosis; score 3, severe lymphocyte depletion with reticular cells or fibrous-tissue proliferation; and score 4, marked follicular atrophy with fibrosis [21].

Serum-neutralizing-antibody titers against IBDV BC6/85 were determined using a microneutralization assay in Douglas Foster-1 (DF-1) cells. Sera were collected from 10 birds per group (*n* = 10), and each serum sample was tested in triplicate in two independent experimental runs. The mean of the two independent runs was used for statistical analysis. Heat-inactivated sera were serially twofold diluted in 10% fetal bovine serum (FBS)-Dulbecco’s Modified Eagle Medium (DMEM) with 1% penicillin and streptomycin antibiotics, mixed with an equal volume containing 100 TCID_50_ of virus and incubated at 37 °C for 1 h. The serum–virus mixtures were subsequently added to DF-1 cell monolayers in 96-well plates. Cell-only, virus-only and serum controls were included, and the plates were incubated at 37 °C, 5% CO_2_ for 4–5 days [22]. The neutralizing-antibody titer was defined as the reciprocal of the highest serum dilution that completely prevented cytopathic effects and was expressed as log_2_ titer.

### 2.7. Vaccine Safety, Absorption and Antibody Persistence

Vaccine absorption was evaluated at necropsy after the 14-day observation period by gross examination of the injection site. Complete absorption was defined as the absence of visible residual vaccine material, oil emulsion, or abnormal tissue changes at the injection site. Partial absorption was defined as limited residual vaccine material with evidence of ongoing clearance, whereas incomplete absorption was defined as obvious persistence of vaccine material or visible residual emulsion. Absorption outcomes were recorded for each individual chicken.

For assessment of antibody persistence, 50 SPF chickens were divided into five groups of 10 and immunized with selected B87 or HLJ0504 unpurified VP2 and VLPs formulations or the commercial attenuated B87 vaccine, as detailed in Table 1. Blood was collected every 2 weeks for 20 weeks, and serum IBDV-specific antibodies were measured by ELISA (IDEXX, Westbrook, ME, USA).

### 2.8. Statistical Analysis

Continuous data (antibody titers, body weight, viral load) were compared among groups using one-way ANOVA followed by Tukey’s multiple comparison test (for all pairwise comparisons) or Dunnett’s test (for comparisons against a single control), with *p* values adjusted for multiplicity. For data collected from the same birds at multiple time points, repeated-measures ANOVA (mixed-effects model in GraphPad Prism 10.1.2) was used, with time as the within-subject factor and treatment as the between-subject factor.

Lesion scores were analyzed using the Kruskal-Wallis test (nonparametric, for ordinal data) followed by Dunn’s multiple comparison test.

Protection rates and vaccine absorption rates were analyzed using Fisher’s exact test (for 2 × 2 comparisons) or chi-square test, as appropriate for categorical outcomes.

All analyses were performed in GraphPad Prism. Statistical significance was defined as *p* < 0.05 (two-tailed), with *p* values adjusted for multiple comparisons where applicable.

## 3. Results

### 3.1. Expression, Purification and VLPs Assembly

**Figure 1:**
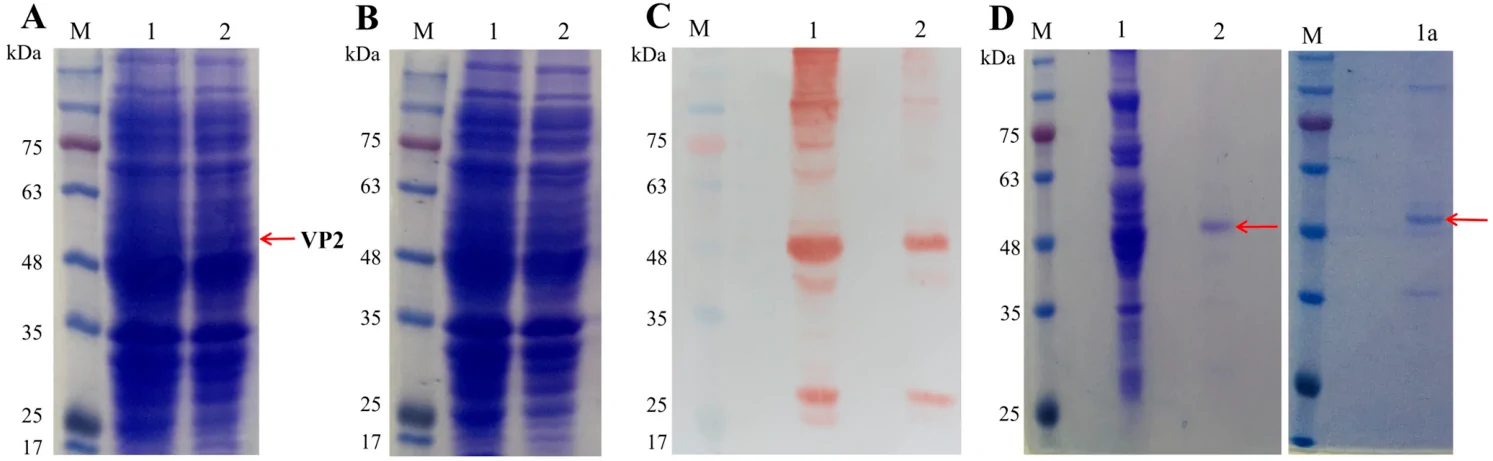
Expression and purification of recombinant IBDV VP2 proteins. (**A**) SDS-PAGE analysis of B87 VP2 expression. Lane M, molecular-weight marker; lane 1, lysate of induced cells expressing B87 VP2; and lane 2, lysate of induced cells carrying the empty vector. (**B**) SDS-PAGE analysis of HLJ0504 VP2 expression. Lane M, molecular-weight marker; lane 1, lysate of induced cells expressing HLJ0504 VP2; and lane 2, lysate of induced cells carrying the empty vector. (**C**) Western blot analysis of recombinant VP2 proteins using an anti-His monoclonal antibody. Lane M, molecular-weight marker; lane 1, lysate of induced cells expressing B87 VP2; and lane 2, lysate of induced cells expressing HLJ0504 VP2. (**D**) SDS-PAGE analysis of VP2 proteins purified by Ni-NTA affinity chromatography followed by sucrose-gradient ultracentrifugation. Lane M, molecular-weight marker; lane 1, unpurified (expressed) B87 VP2; lane 2, purified B87 VP2 protein; and lane 1a, purified HLJ0504 VP2 protein. The original image can be found in Supplementary Material File S1.

**Figure 2:**
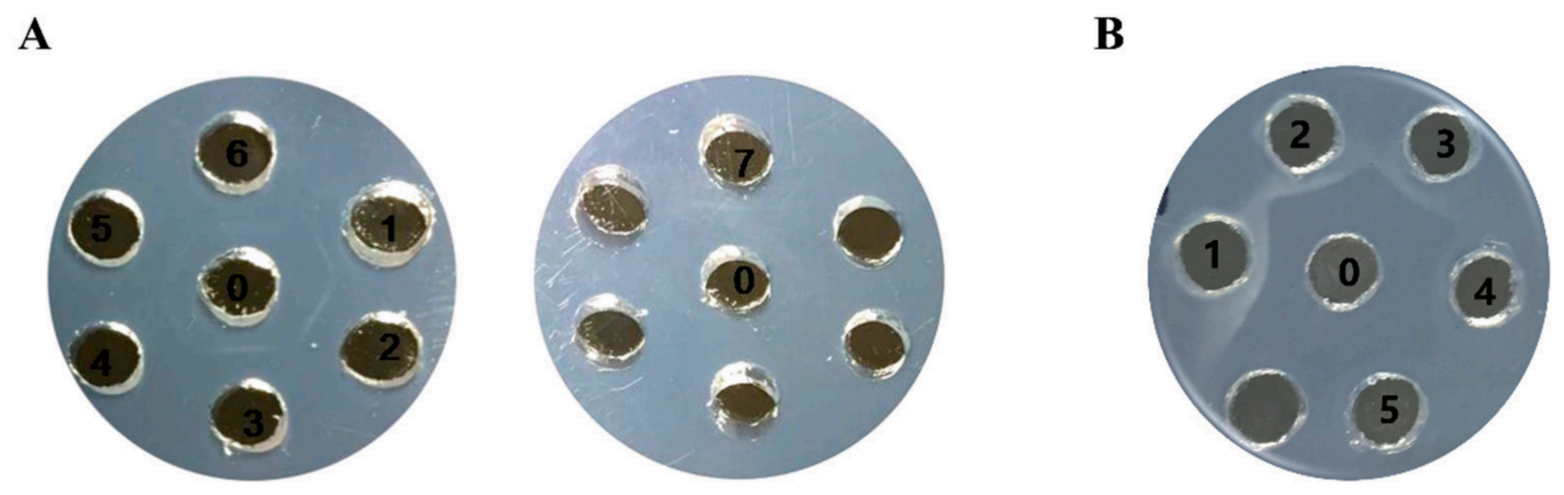
Agar-gel precipitation of unpurified recombinant VP2 proteins. (**A**) AGP of the unpurified B87 VP2 preparation. Well 0, reference IBDV antiserum; well 1, reference IBDV antigen; and wells 2–7, recombinant bacterial lysate diluted 1:2, 1:4, 1:8, 1:16, 1:32, and 1:64, respectively. (**B**) AGP of the unpurified HLJ0504 VP2 preparation. Well 0, reference IBDV antiserum; well 1, reference IBDV antigen; and wells 2–5, recombinant bacterial lysate diluted 1:2, 1:4, 1:8, and 1:16, respectively.

Recombinant VP2 proteins from the IBDV B87 and HLJ0504 strains were detected as bands of approximately 50 kDa by SDS-PAGE and confirmed by anti-His W.B (Figure 1A–C). Following Ni-NTA chromatography and sucrose-gradient ultracentrifugation, B87 and HLJ0504 VP2 reached purities of >90% and approximately 70%, with concentrations of 0.52 and 0.14 mg mL^−1^, respectively (Figure 1D). Both soluble proteins retained antigenicity, producing agar-gel precipitation titers of 1:64 for B87 and 1:16 for HLJ0504 (Figure 2).

**Figure 3:**
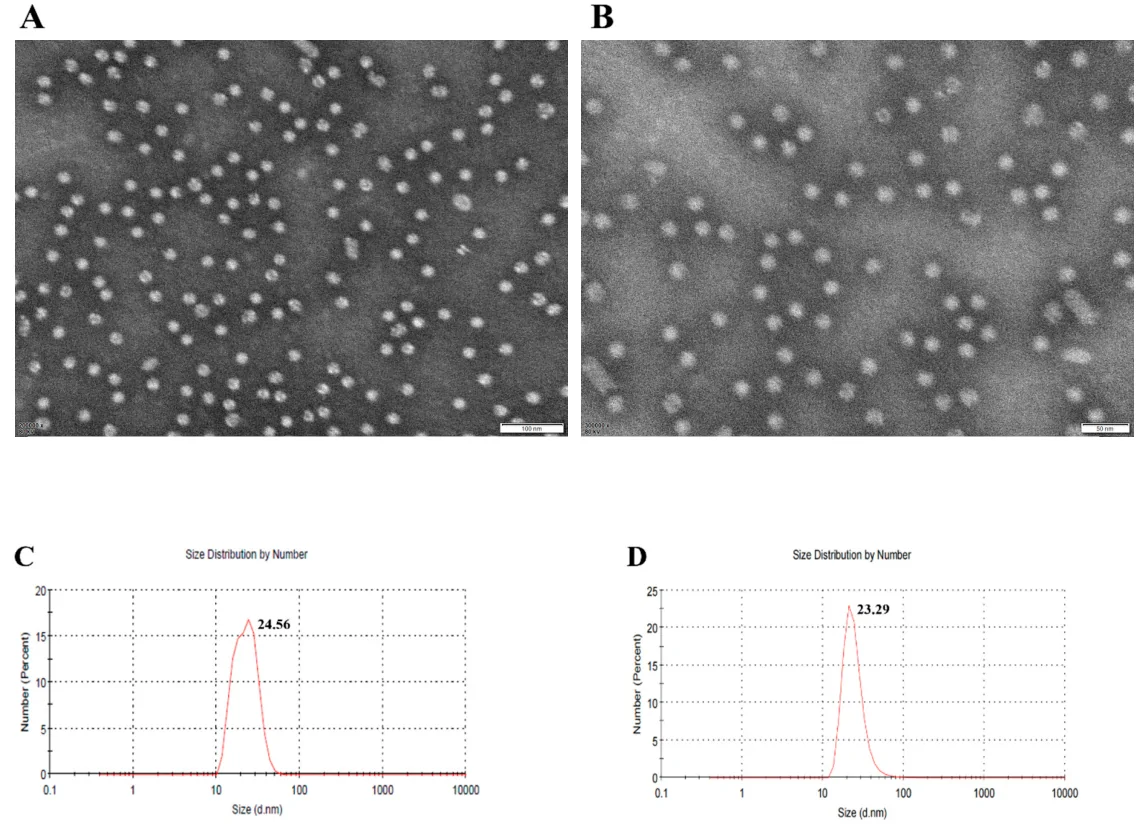
Morphological characterization and size—distribution analysis of VP2 VLPs. (**A**) TEM image of VLPs assembled from purified B87 VP2 protein in PBS. (**B**) TEM image of VLPs assembled from purified HLJ0504 VP2 protein in PBS. Scale bar, 200 nm. Enlarged regions show VLPs with diameters of approximately 25 nm. (**C**) DLS size-distribution profile of B87 VP2 VLPs. (**D**) DLS size-distribution profile of HLJ0504 VP2 VLPs.

TEM showed that purified VP2 proteins self-assembled into VLPs (Figure 3A,B). DLS indicated relatively uniform particle distributions with diameters of approximately 25 nm (Figure 3C,D). Fewer particles were observed in the HLJ0504 preparation, consistent with their lower protein concentration and purity.

### 3.2. Serum-Antibody Responses

Antibody responses to the experimental subunit and commercial inactivated vaccines increased markedly between weeks 2 and 4, whereas responses to the commercial attenuated vaccine increased earlier and subsequently declined.

**Figure 4:**
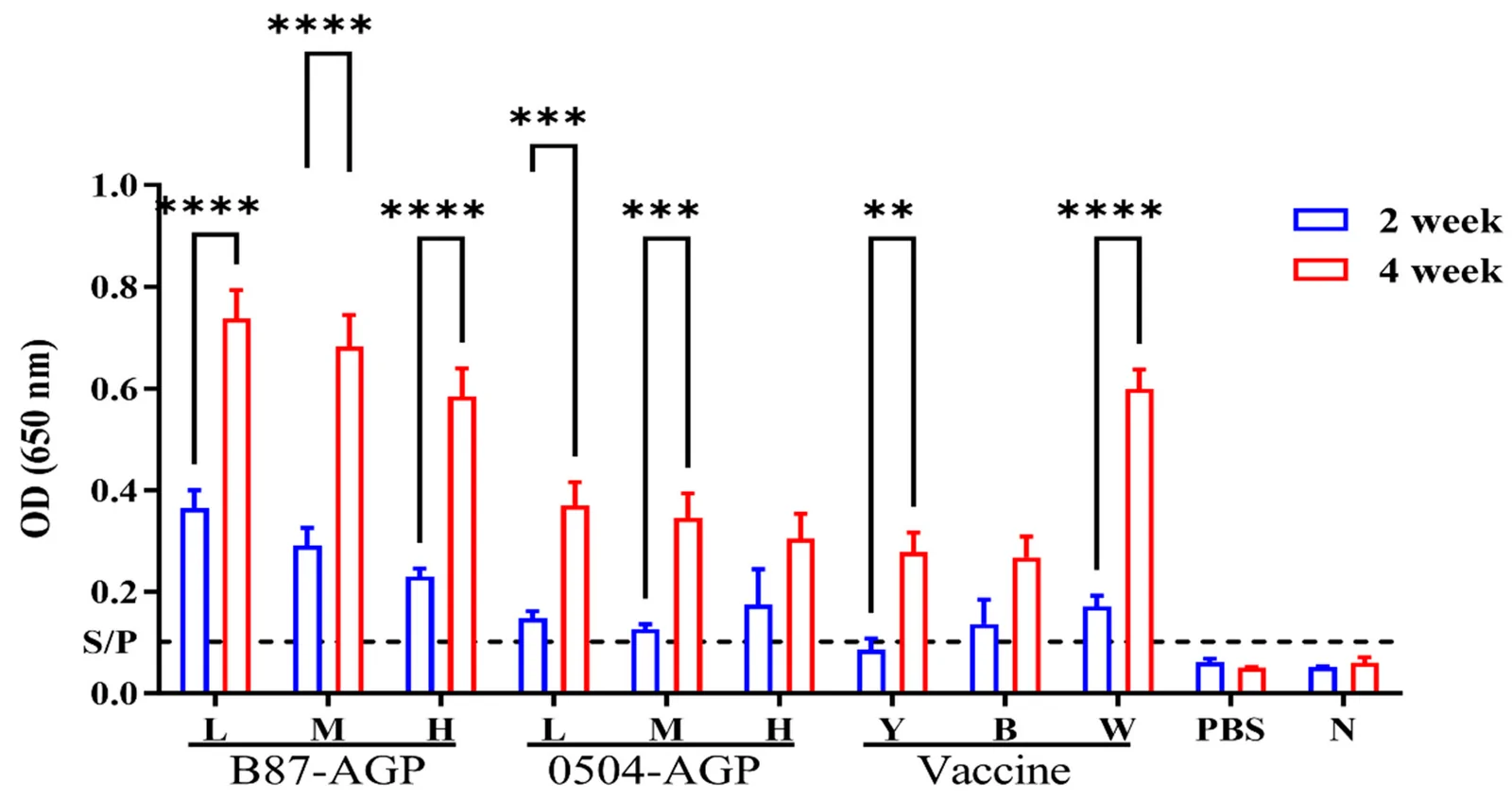
Serum IBDV-specific antibody responses induced by unpurified VP2 formulations. Serum-antibody levels were determined by ELISA. B87-AGP, subunit vaccine containing unpurified B87 VP2 protein quantified by AGP; B87-AGP (L), AGP titer of 1:10; B87-AGP (M), AGP titer of 1:20; B87-AGP (H), AGP titer of 1:40. 0504-AGP, subunit vaccine containing unpurified HLJ0504 VP2 protein quantified by AGP; 0504-AGP (L), AGP titer of 1:10; 0504-AGP(M), AGP titer of 1:20; and 0504-AGP (H), AGP titer of 1:40. Vaccine (Y), commercial subunit vaccine with an AGP titer of at least 1:16; Vaccine (B), commercial attenuated B87 vaccine; Vaccine (W), commercial inactivated vaccine; PBS, PBS control; and N, negative control. ** *p* < 0.01, *** *p* < 0.001, and **** *p* < 0.0001.

Among the unpurified formulations, day-28 ELISA values decreased with increasing antigen dilution. Values for B87 VP2 were 0.74, 0.68 and 0.58 at agar-gel precipitation titers of 1:10, 1:20 and 1:40, respectively; corresponding values for HLJ0504 VP2 were 0.39, 0.35 and 0.31 (Figure 4).

**Figure 5:**
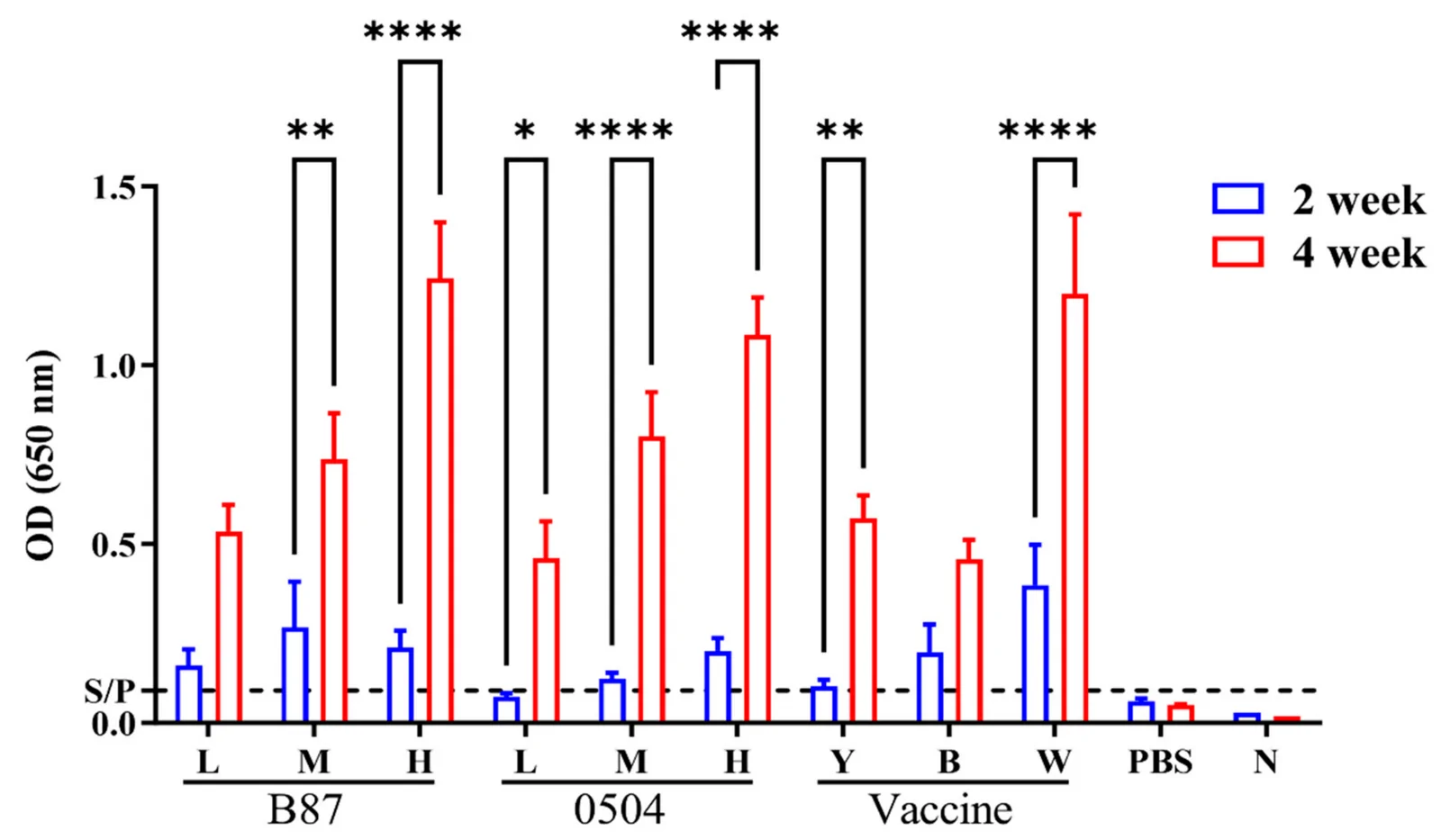
Serum IBDV-specific antibody responses induced by purified VP2 VLPs formulations. Serum-antibody levels were determined by ELISA. B87, subunit vaccine containing VLPs assembled from purified B87 VP2 protein; B87 (L), 1 µg of VLPs antigens; B87 (M), 5 µg of VLPs antigens; and B87 (H), 10 µg of VLPs antigens. 0504, subunit vaccine containing VLPs assembled from purified HLJ0504 VP2 protein; 0504 (L), 1 µg of VLPs antigens; 0504 (M), 5 µg of VLPs antigens; and 0504 (H), 10 µg of VLPs antigens. Vaccine (Y), commercial subunit vaccine with an AGP titer of at least 1:16; Vaccine (B), commercial attenuated B87 vaccine; Vaccine (W), commercial inactivated vaccine; PBS, PBS control; and N, negative control. * *p* < 0.05, ** *p* < 0.01, and **** *p* < 0.0001.

**Figure 6:**
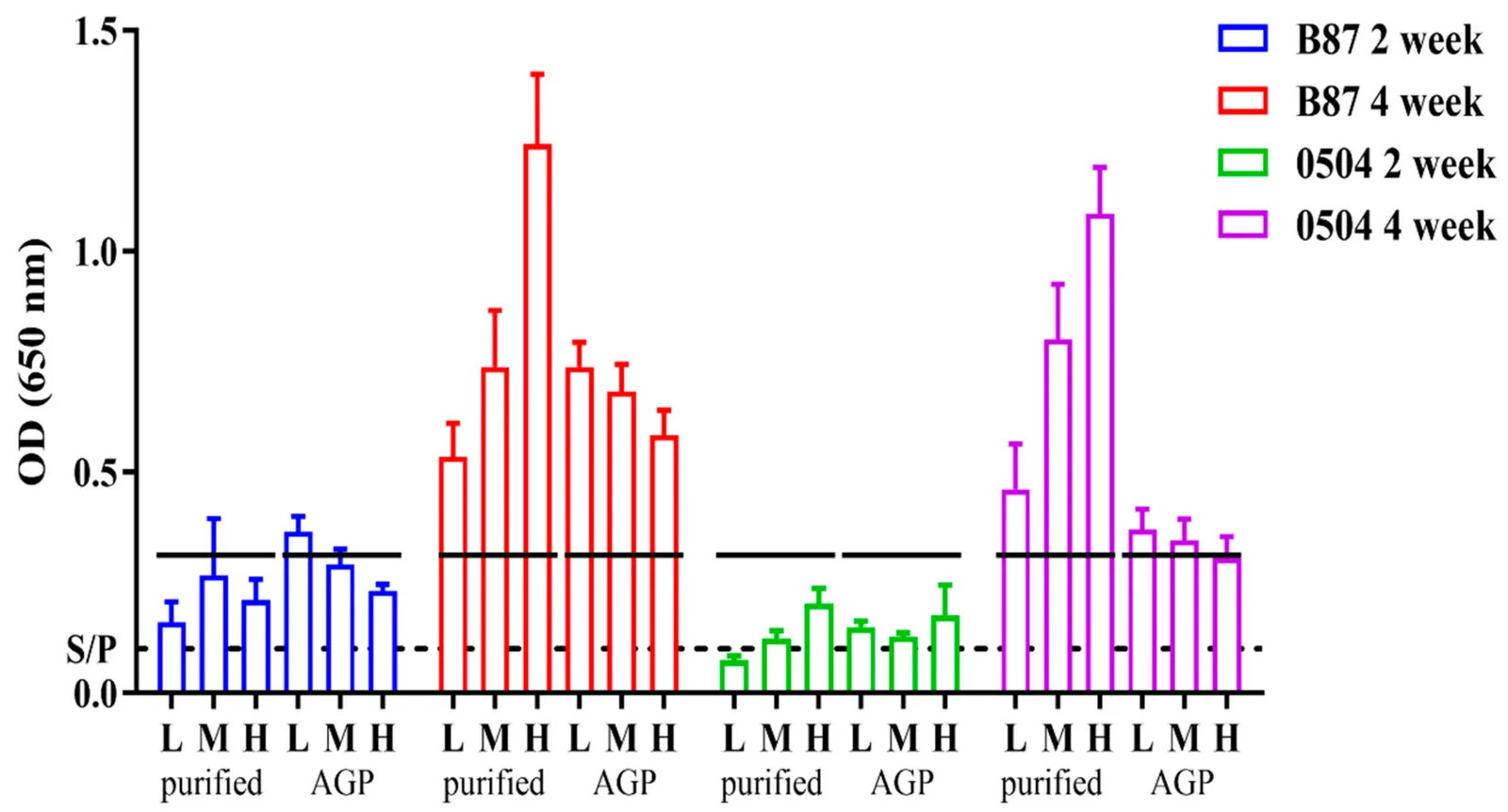
Comparison of serum-antibody responses induced by purified VLPs and unpurified VP2 formulations. Purified, subunit vaccines containing VLPs assembled from purified VP2 protein; Purified (L), 1 µg of VLPs antigens; Purified (M), 5 µg of VLPs antigens; and Purified (H), 10 µg of VLPs antigens. AGP, subunit vaccines containing unpurified VP2 protein; AGP (L), AGP titer of 1:10; AGP (M), AGP titer of 1:20; and AGP (H), AGP titer of 1:40.

Purified VLPs induced dose-dependent responses. Day-28 ELISA values for B87 VLPs were 0.54, 0.74 and 1.24 at doses of 1, 5 and 10 µg, respectively, compared with 0.46, 0.80 and 1.09 for HLJ0504 VLPs (Figure 5). The commercial inactivated, subunit and attenuated vaccines produced values of 1.20, 0.57 and 0.49, respectively. Overall, B87-derived formulations induced higher antibody responses than corresponding HLJ0504 formulations. Under the tested formulation conditions, purified VP2 VLPs preparations generally produced stronger antibody responses than unpurified VP2 preparations; however, these differences should be interpreted considering the distinct antigen normalization strategies used for the two vaccine formats (Figure 6).

Overall comparison of the antibody responses at weeks 2 and 4 showed that formulations derived from the B87 strain induced higher antibody levels than the corresponding formulations derived from the HLJ0504 strain. In addition, purified VLP formulations generally induced higher antibody responses than the corresponding unpurified VP2 formulations (Figure 6).

### 3.3. HLJ0504 VP2 Induces Higher Neutralizing-Antibody Responses than B87 VP2 Responses

**Figure 7:**
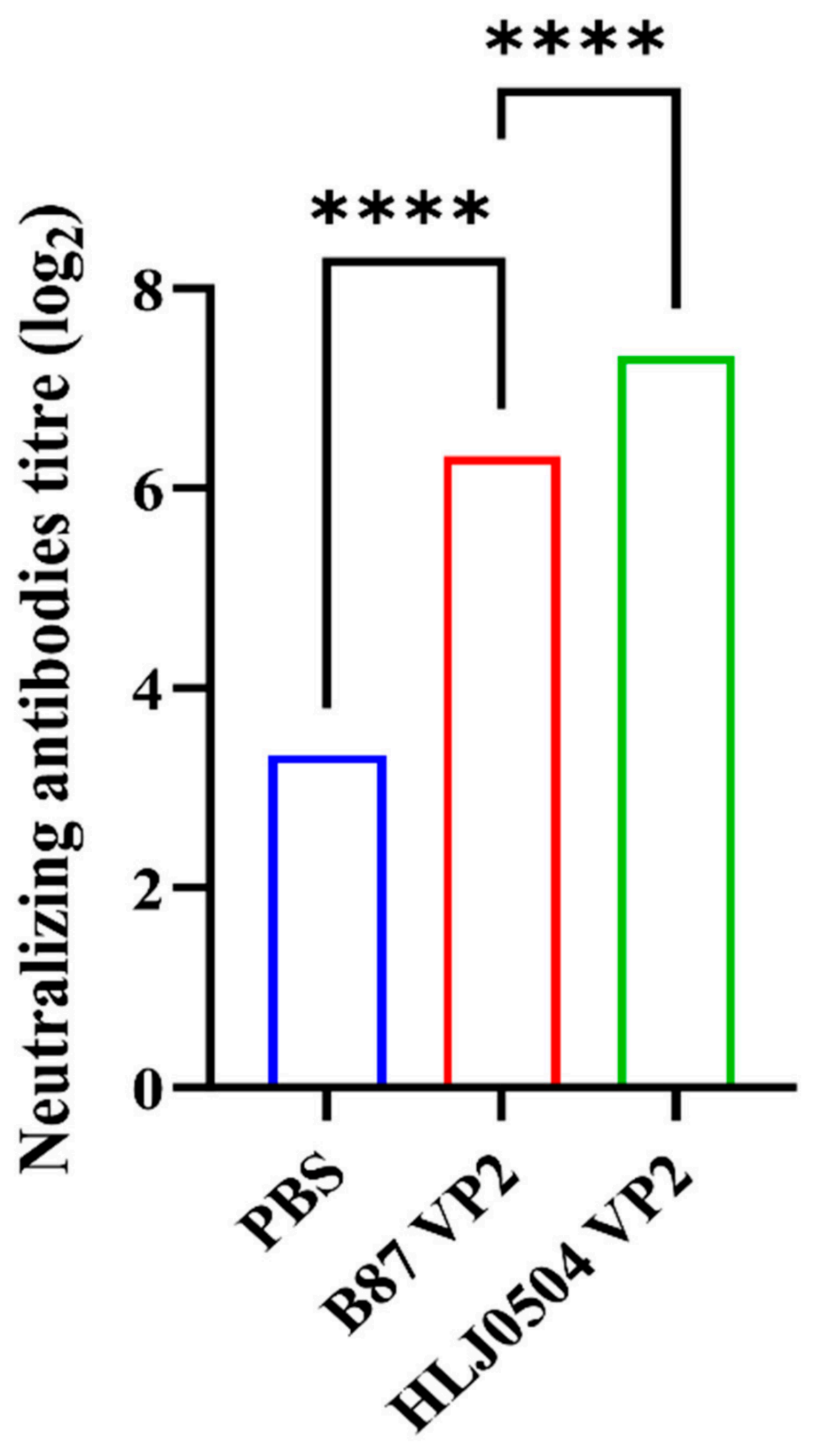
Serum-neutralizing-antibody responses induced by B87 and HLJ0504 VLPs. Neutralizing-antibody titers were determined in the PBS, B87 VP2 and HLJ0504 VP2 groups and expressed on a log_2_ scale. Serum-neutralizing-antibody titers against IBDV in chickens immunized with the indicated vaccines at 28 days post-vaccination. Data are presented as mean ± SD (*n* = 10 per group). Statistical analysis was performed using one-way ANOVA with Tukey’s multiple comparison test, **** *p* < 0.0001.

Serum-neutralizing-antibody titers increased from the PBS group to the B87 VP2 group and were highest in the HLJ0504 VP2 group. B87 VP2 significantly increased neutralizing-antibody titers compared with PBS, whereas HLJ0504 VP2 produced a further significant increase compared with B87 VP2 (*p* < 0.0001 for both comparisons) (Figure 7).

### 3.4. Protective Efficacy Against Virulent IBDV

**Figure 8:**
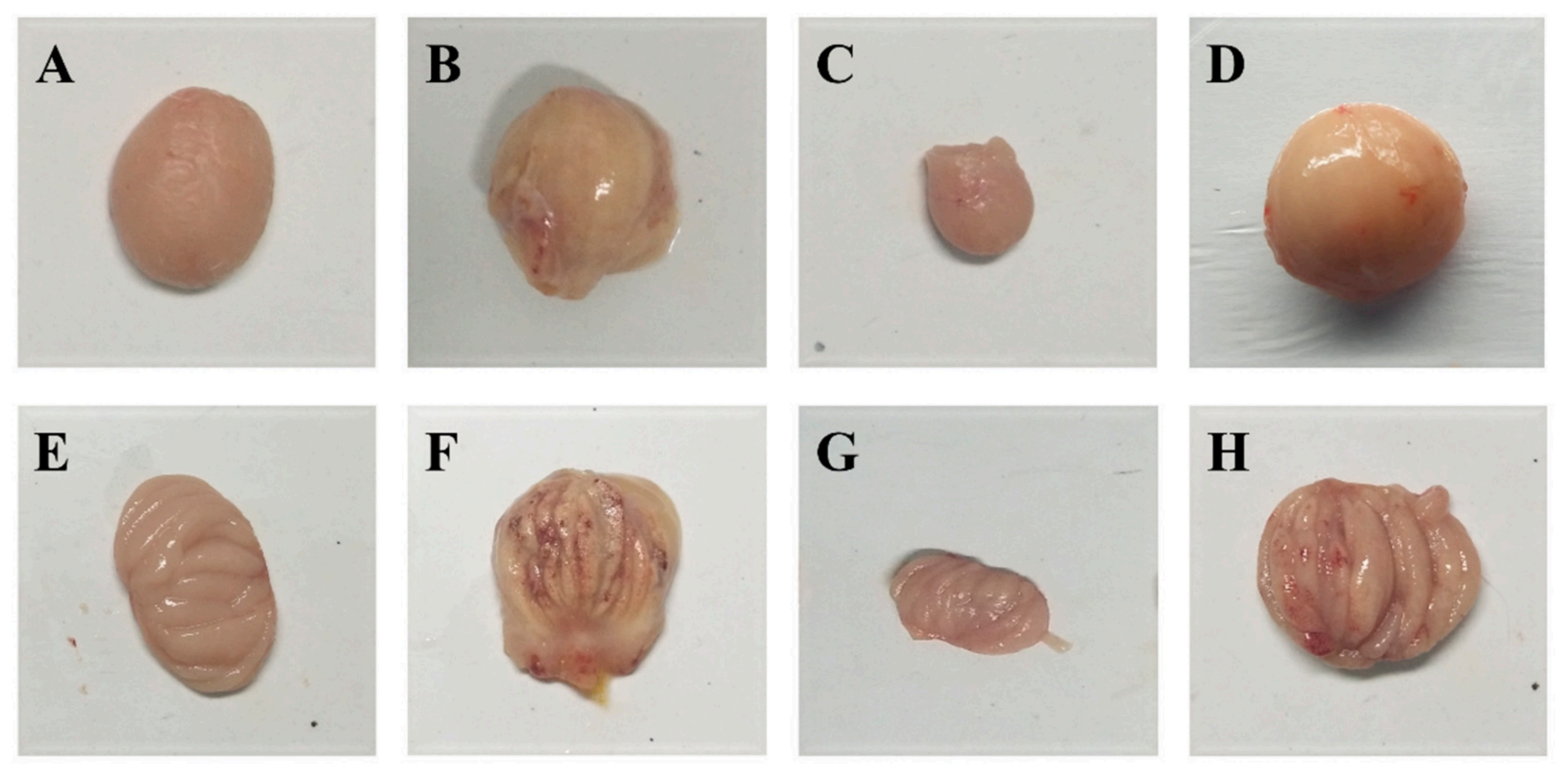
Gross lesions of the bursae of Fabricius following challenge with virulent IBDV strain BC6/85. (**A**,**E**) Normal bursae from unvaccinated and unchallenged chickens. (**B**,**F**) Yellow, gelatinous, and hemorrhagic bursae from challenged chickens. (**C**,**G**) Atrophic bursae from chickens vaccinated with the attenuated vaccine. (**D**,**H**) Swollen bursae from challenged chickens.

No mortality occurred during the 4-day observation period after challenge with virulent IBDV BC6/85. Unvaccinated challenged chickens developed characteristic lesions, including muscular hemorrhage, bursal swelling or atrophy, gelatinous exudation and bursal hemorrhage (Figure 8).

**Figure 9:**
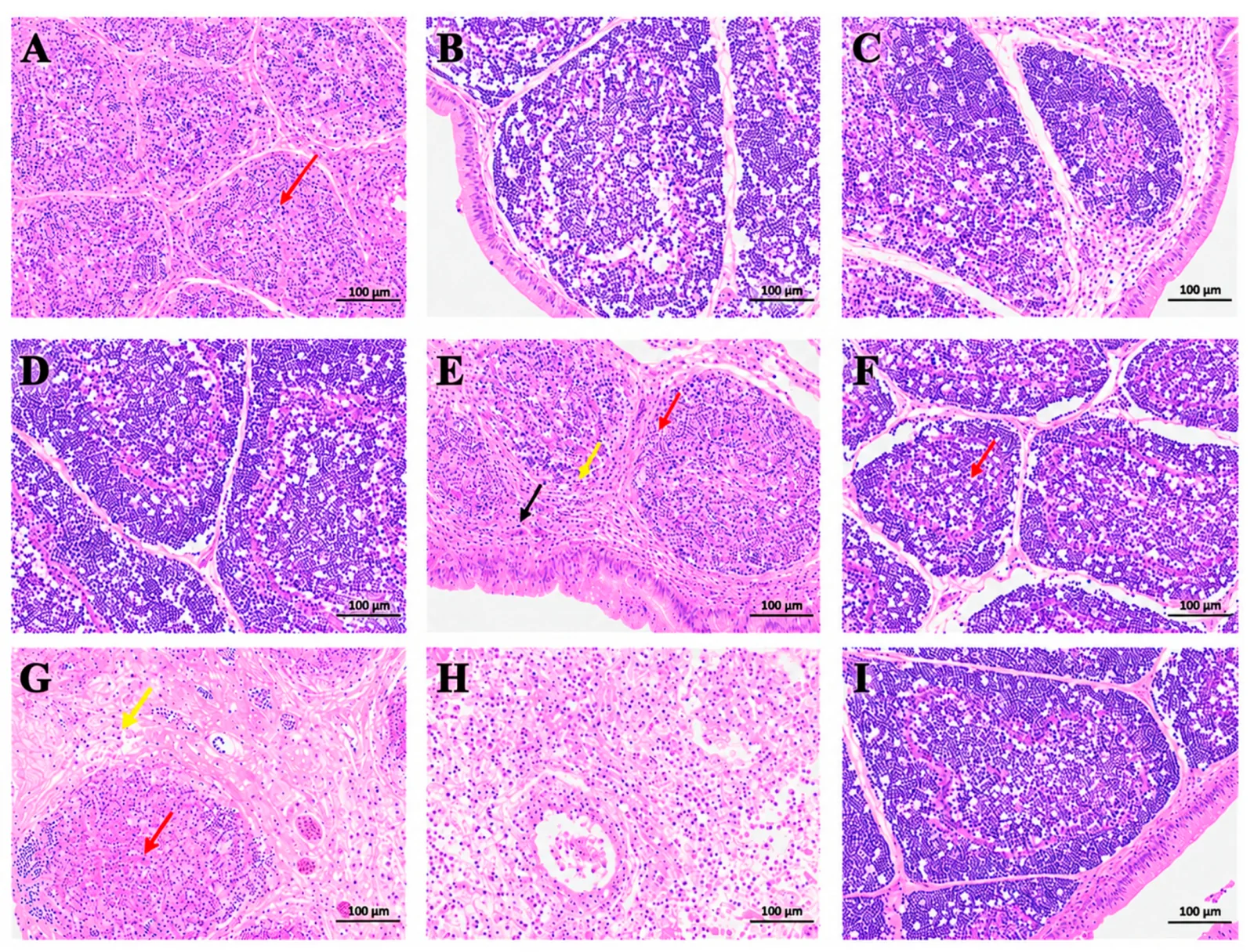
Histopathological changes in the bursae of Fabricius following challenge with virulent IBDV. Representative H&E-stained bursal sections from chickens vaccinated with (**A**) B87 VP2 at an AGP titer of 1:20, (**B**) B87 VLPs at 10 µg, (**C**) HLJ0504 VP2 at an AGP titer of 1:10, (**D**) HLJ0504 VLPs at 10 µg, (**E**) commercial subunit vaccine, (**F**) commercial inactivated vaccine, or (**G**) commercial attenuated vaccine; (**H**) unvaccinated chickens following challenge with virulent IBDV strain BC6/85; and (**I**) unvaccinated and unchallenged chickens. Sections were stained with H&E and examined at ×200 magnification. Black arrows indicate increased bursal epithelial cells accompanied by mild heterophilic granulocyte infiltration. Red arrows indicate follicular-cell necrosis and abundant disintegrating cellular debris within the follicular medulla. Yellow arrows indicate partial loss of follicular architecture accompanied by inflammatory-cell infiltration and exudation.

Among the unpurified formulations, B87 VP2 at an agar-gel precipitation titer of 1:20 protected 8/10 chickens, whereas HLJ0504 VP2 at 1:10 protected 9/10. Among the purified formulations, 10 µg of B87 and HLJ0504 VLPs protected 8/10 and 9/10 chickens, respectively. The commercial inactivated, subunit and attenuated vaccines protected 9/10, 7/10 and 5/10 chickens, respectively (Table 2). As shown in Figure 9, VP2 VLPs formulations induced variable levels of protection depending on antigen dose and formulation conditions.

Histopathological examination showed mild lymphocyte depletion, focal necrosis and inflammatory-cell infiltration in chickens receiving B87 VP2 at 1:20 or the commercial subunit vaccine. In contrast, chickens immunized with 10 µg of B87 VLPs, HLJ0504 VP2 at 1:10, 10 µg of HLJ0504 VLPs or the commercial inactivated vaccine largely retained normal bursal architecture. Severe follicular necrosis, cellular debris accumulation, inflammatory infiltration and loss of follicular organization were observed in the challenged control and commercial attenuated-vaccine groups (Figure 9).

### 3.5. Safety and Local Absorption

**Figure 10:**
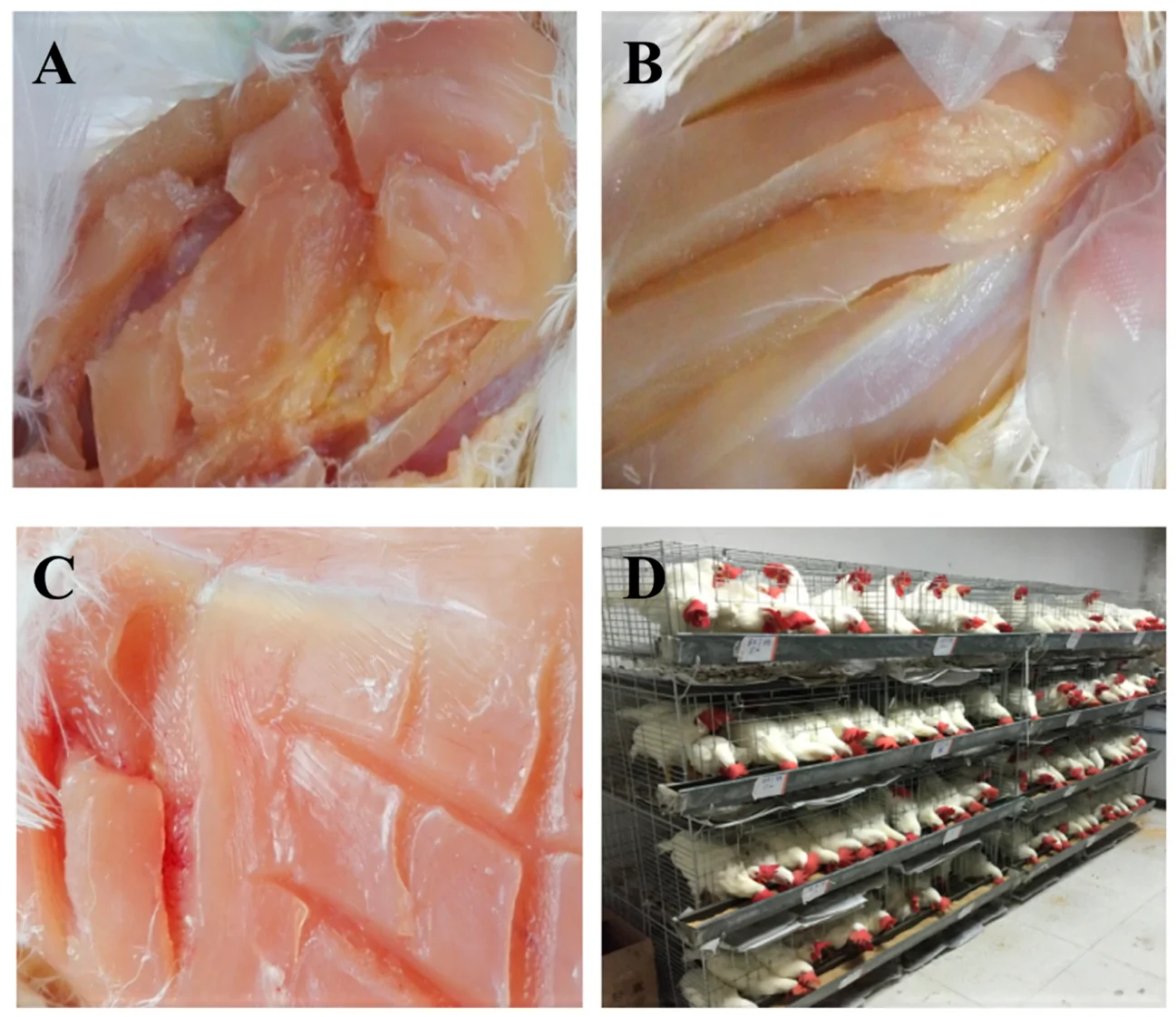
Gross evaluation of vaccine absorption at injection sites. (**A**) Incomplete absorption following administration of a 1 mL vaccine dose. (**B**) Partial absorption following administration of a 0.5 mL vaccine dose. (**C**) Effective absorption following administration of a 0.25 mL vaccine dose. (**D**) Representative chickens showing normal activity and feed intake following vaccination.

No systemic adverse reactions, injection-site inflammation or nodules were observed during the 14-day safety assessment. Vaccine absorption decreased as the administration volume increased and was generally greater for purified VLPs than for unpurified VP2 preparations (Table 3, and Figure 10).

Complete absorption varied according to formulation type and administration volume. For example, complete absorption of B87 VLPs was observed on 8/10, 8/10, and 3/10 chickens receiving 250, 500, and 1000 µL, respectively, whereas corresponding rates for unpurified B87 VP2 preparations were lower. Similar volume-dependent trends were observed for HLJ0504 formulations. These findings indicate that lower administration volumes were associated with improved local vaccine clearance under the conditions evaluated; however, the results should be interpreted as descriptive observations because the study was not powered for formal statistical comparison of absorption outcomes.

### 3.6. Persistence of Serum Circulating Antibodies

**Figure 11:**
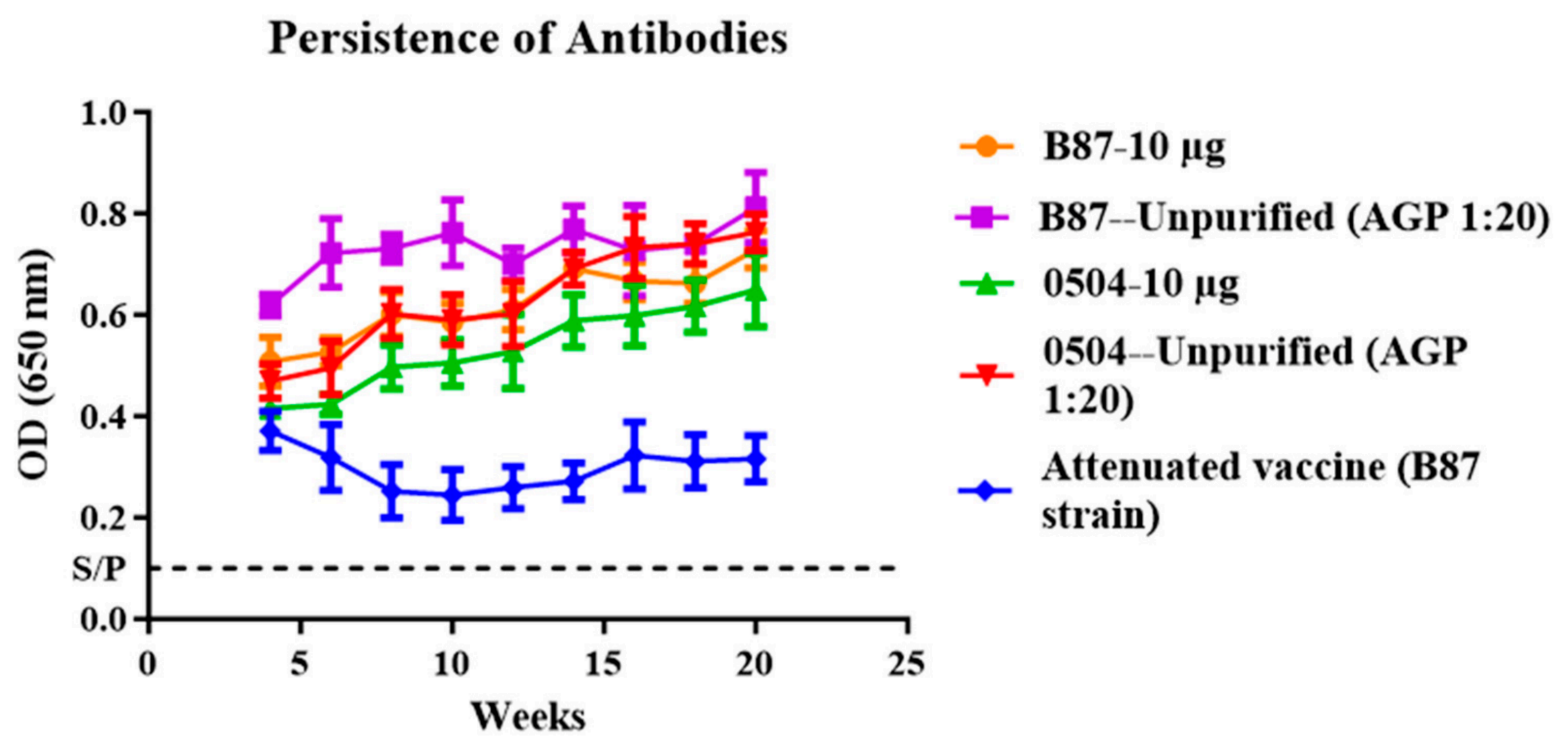
Persistence of serum-antibody responses following VP2 immunization. Serum IBDV-specific antibody levels were monitored after immunization with recombinant VP2 formulations. Antibody responses increased after vaccination, reached maximal levels at approximately week 4, and remained detectable throughout the 20-week observation period. These data represent persistence of circulating antibody responses. “S/P" represents the Serum-to-Positive control (S/P) cut-off value.

All selected recombinant VP2 and VLPs formulations produced sustained serum-antibody responses over the 20-week observation period. Antibody levels increased following immunization, peaked at approximately week 4, and remained elevated throughout the monitoring period. These findings demonstrate persistence of vaccine-induced humoral responses but do not directly establish long-term protective immunity, which would require challenge studies performed at later time points (Figure 11).

## 4. Discussion

This study demonstrates that recombinant VP2 proteins derived from the IBDV B87 and HLJ0504 strains can be successfully expressed in *Escherichia coli*, retain antigenic activity, self-assemble into VLPs, and induce substantial protection against virulent IBDV challenge [23]. Although the two VP2 proteins displayed differences in expression yield and purification efficiency, both formed relatively uniform particles of approximately 25 nm. These findings support the suitability of VP2 as a self-assembling antigen for the development of non-replicating IBDV subunit vaccines [11].

The B87 VP2 preparation exhibited higher purity, protein concentration, and agar-gel precipitation activity than the HLJ0504 preparation. The stronger AGP titer of B87 VP2 indicates that this recombinant protein retained comparatively greater detectable antigenic activity following bacterial expression [9]. However, the lower yield and purity of HLJ0504 VP2 did not prevent particle assembly or protective immunogenicity [24]. This observation indicates that production yield and AGP reactivity alone may not fully determine the biological performance of a vaccine antigen. Optimization of expression, purification, and particle recovery may nevertheless improve the consistency and scalability of the HLJ0504 VLPs preparation [8].

Purified VP2 VLPs formulations generally induced stronger antibody responses than the corresponding unpurified VP2 preparations under the experimental conditions evaluated [25]. However, this comparison should be interpreted cautiously because the two antigen formats were standardized using different approaches. Unpurified VP2 preparations were normalized according to AGP titers, whereas purified VP2 VLPs formulations were administered based on VP2 protein concentration; therefore, equivalent VP2 antigen exposure between these groups cannot be assumed. Furthermore, crude bacterial preparations may contain residual bacterial-derived components that could contribute to immune stimulation independently of VP2 [26]. Thus, although the enhanced immune responses observed with purified VP2 VLPs may be associated with improved antigen presentation and particulate organization, the individual contributions of purification, VLPs assembly, and antigen dose require further investigation using directly comparable antigen quantities and formulations [8].

The temporal antibody profiles also differed among vaccine types. The commercial attenuated B87 vaccine induced a relatively rapid response by week 2, followed by a reduction at week 4. In contrast, antibody responses to the experimental subunit vaccines developed more gradually and increased markedly between weeks 2 and 4. More importantly, the selected purified VLPs and unpurified VP2 formulations maintained elevated antibody levels throughout the 20-week observation period, whereas the response to the attenuated vaccine declined after week 4. These findings indicate that the experimental vaccines can induce durable humoral responses [13]. Nevertheless, the prolonged persistence of serum antibodies indicates sustained humoral immunogenicity following VP2 vaccination; however, antibody persistence alone cannot be interpreted as equivalent to prolonged protective immunity. Protective duration requires confirmation through challenge experiments performed at defined later time points, together with evaluation of additional immune correlates, including cellular immune responses and functional antibody activity.

Protection against virulent BC6/85 challenge further demonstrated the biological activity of the experimental vaccines, similar to the study of Chen et al. [9]. The optimal HLJ0504 formulations, including 10 µg of purified VLPs and unpurified VP2 at an AGP titer of 1:10, showed protection in nine of 10 chickens, while the corresponding B87 formulations protected eight of 10 chickens. These observations indicate comparable protective trends among the optimized VP2 formulations and commercial vaccine controls under the conditions evaluated; however, the relatively small group size limits definitive conclusions regarding superiority among formulations [24].

An important finding was that the magnitude of the ELISA antibody response did not completely correspond to the observed protection rate [27]. As described in [28], our findings show that B87-derived formulations generally induced higher binding-antibody levels than HLJ0504 formulations, yet the optimal HLJ0504 vaccines provided slightly greater protection, indicating that antibody quantity alone was not sufficient to predict efficacy. In the complementary VNT assay, both VP2 antigens elicited functional neutralizing antibodies, with HLJ0504 VP2 producing the highest titer and significantly exceeding B87 VP2 and PBS (*p* < 0.0001). These findings indicate that HLJ0504 VP2 possesses greater neutralizing immunogenicity than B87 VP2 [22].

Gross and histopathological examinations supported the protection results. Chickens vaccinated with 10 µg of B87 VLPs, 10 µg of HLJ0504 VLPs, HLJ0504 VP2 at an AGP titer of 1:10, or the commercial inactivated vaccine largely retained normal bursal architecture after challenge. In contrast, severe follicular necrosis, inflammatory-cell infiltration, cellular debris accumulation, and loss of follicular organization were observed in unvaccinated challenged chickens and in several chickens receiving the commercial attenuated vaccine. Preservation of the bursa of Fabricius (BF) is particularly relevant because bursal damage is a central pathological consequence of IBDV infection [29]. Therefore, the experimental vaccines not only reduced gross disease but also limited tissue-level injury in the principal target organ.

All experimental formulations were well tolerated during the 14-day safety evaluation. No systemic adverse reactions, injection-site inflammation, or nodules were detected. The present findings indicate that vaccine absorption was influenced by both formulation type and administration volume, as reported in other studies [30]. Purified VLPs formulations generally showed better absorption than unpurified VP2 preparations, while increasing the injection volume from 250 to 1000 µL substantially reduced complete absorption [31]. The HLJ0504 VLPs formulation showed complete absorption in all chickens receiving the 250 µL dose, whereas only four of 10 chickens showed complete absorption at 1000 µL. These results support the use of lower administration volumes and identify 0.25 mL as the most suitable volume for young chickens, while 0.5 mL may be considered for adult birds.

Although differences in absorption rates were observed among formulations and administration volumes, these findings should be interpreted cautiously. The absorption assessment was based on gross examination at a single necropsy time point and was intended primarily as a safety and formulation characterization endpoint [32]. Because the experimental groups were relatively small and no formal statistical comparison of absorption proportions was performed, these observations indicate trends in local clearance rather than definitive differences in absorption kinetics. Future studies using larger cohorts, quantitative residual-material measurements, and repeated sampling time points would provide a more comprehensive evaluation of vaccine absorption.

Several limitations should be considered when interpreting the protective efficacy results. First, the challenge experiment was conducted using relatively small groups of SPF chickens (generally *n* = 10 per group), which limits the statistical power for detecting small differences between formulations. Therefore, numerical differences in protection rates, particularly comparisons involving one or two birds, should be interpreted cautiously and considered as trends rather than definitive evidence of superiority. Second, protection was evaluated using a single virulent IBDV BC6/85 challenge strain and therefore may not fully represent protection against genetically diverse field strains. Third, the post-challenge observation period was limited to four days, and protection was primarily assessed based on clinical observations, gross bursal lesions, and histopathological changes. Future studies involving larger-scale vaccination trials, diverse IBDV challenge strains, longer observation periods, and additional immune correlates will be necessary to further validate the breadth and durability of protection provided by VP2-based vaccine formulations [33].

## 5. Conclusions

Overall, the results identify recombinant B87 and HLJ0504 VP2 vaccines as promising alternatives to conventional IBDV vaccines. Purified VLPs formulations provided strong and sustained antibody responses, substantial protection, preservation of bursal architecture, favorable absorption, and an acceptable safety profile, while the complementary VNT assay confirmed higher functional neutralization by HLJ0504 VP2 than by B87 VP2. Among the tested candidates, the 10-µg HLJ0504 VLPs formulation showed a particularly balanced performance, achieving 90% protection and complete absorption at the 250 µL administration volume. These findings provide a strong experimental basis for further development and optimization of VP2 VLPs-based vaccines for the prevention of infectious bursal disease.

## Figures and Tables

**Table 1:** Experimental design for antibody monitoring period.

Group	Composition	Immunizing Dose	Administration	Number of Chickens	Age (Days) at Vaccination	Immune Duration
A	B87 VP2 (AGP 1:20)	250 µL	Intramuscular	10	28	20 weeks
B	B87 VLPs (10 μg)			10		
C	0504 VP2 (AGP 1:20)			10		
D	0504 VLPs (10 μg)			10		
E	Attenuated vaccine (B87 strain)	1.5 feathers/1 mL	Oral	10		

**Table 2:** Protection efficacy against virulent IBDV BC6/85 strain.

Group	Vaccine	Animals with BF Lesions ^a^	Animals with BF/BW Ratio <2 ^b^	Histopathological BF Lesion Score ^c^				Protection ^d^
				1	2	3	4	
A	B87 VP2 (AGP 1:10)	3/10	1/10	7	3	0	0	7/10
B	B87 VP2 (AGP 1:20)	2/10	0/10	8	2	0	0	8/10
C	B87 VP2 (AGP 1:40)	6/10	0/10	4	3	2	1	4/10
D	0504 VP2 (AGP 1:10)	1/10	0/10	9	1	0	0	9/10
E	0504 VP2 (AGP 1:20)	4/10	0/10	6	3	1	0	6/10
F	0504 VP2 (AGP 1:40)	4/10	0/10	6	4	0	0	6/10
G	B87 VLPs (1 μg)	5/10	0/10	5	3	1	1	5/10
H	B87 VLPs (5 μg)	3/10	0/10	7	2	1	0	7/10
I	B87 VLPs (10 μg)	2/10	0/10	8	2	0	0	8/10
J	0504 VLPs (1 μg)	5/10	0/10	5	1	1	3	5/10
K	0504 VLPs (5 μg)	2/10	0/10	8	2	0	0	8/10
L	0504 VLPs (10 μg)	1/10	0/10	9	1	0	0	9/10
M	Subunit vaccine	3/10	0/10	7	3	0	0	7/10
N	Inactivated vaccine	1/10	1/10	9	1	0	0	9/10
O	Attenuated vaccine	5/10	4/10	5	2	1	2	5/10
P	Challenged control	10/10	0/10	0	0	2	8	0/10
Q	PBS	0/10	0/10	10	0	0	0	10/10
R	-	0/5	0/5	5	0	0	0	5/5

^a^ BF, bursa of Fabricius; BF/BW, bursa-to-body-weight ratio. The gross-lesion results are expressed as the number of birds with severe bursal lesions relative to the total number examined. ^b^ A BF/BW ratio below 2 was considered indicative of bursal atrophy. ^c^ Histopathological lesion scores were assigned according to the criteria described in the Methods. ^d^ Protection is expressed as the number of protected chickens relative to the total number of chickens in each group. B87 denotes the B87 strain, and 0504 denotes the HLJ0504 strain.

**Table 3:** Safety testing results of vaccine.

Group	Composition	Immunizing Dose	Injection Site	Number of Chickens	Days of Observation	Test Result		
						Local Reaction	Systemic Reaction	Vaccine Absorption Rate
B87 VLPs	10 μg	250 μL	Pectoral muscle	10	14	—	—	8/10
B87 VLPs	10 μg	500 μL	Pectoral muscle	10	14	—	—	8/10
B87 VLPs	10 μg	1000 μL	Pectoral muscle	10	14	—	—	3/10
B87 VP2	AGP 1:20	250 μL	Pectoral muscle	10	14	—	—	8/10
B87 VP2	AGP 1:20	500 μL	Pectoral muscle	10	14	—	—	5/10
B87 VP2	AGP 1:20	1000 μL	Pectoral muscle	10	14	—	—	1/10
0504 VLPs	10 μg	250 μL	Pectoral muscle	10	14	—	—	10/10
0504 VLPs	10 μg	500 μL	Pectoral muscle	10	14	—	—	8/10
0504 VLPs	10 μg	1000 μL	Pectoral muscle	10	14	—	—	4/10
0504 VP2	AGP 1:20	250 μL	Pectoral muscle	10	14	—	—	7/10
0504 VP2	AGP 1:20	500 μL	Pectoral muscle	10	14	—	—	5/10
0504 VP2	AGP 1:20	1000 μL	Pectoral muscle	10	14	—	—	2/10

## Data Availability

The original contributions presented in this study are included in the article/Supplementary Material. Further inquiries can be directed to the corresponding authors.

## Supplementary Materials

The following supporting information can be downloaded at: https://www.mdpi.com/article/10.3390/vetsci13101033/s1, Table S1: Experimental design for chicken immunization. File S1: Original image.

## Abbreviations

The following abbreviations are used in this manuscript:

AGP	Agar-gel precipitation
BF	Bursa of Fabricius
BID	Bursa infective dose
DF-1	Douglas Foster-1
DLS	Dynamic light scattering
DMEM	Dulbecco’s modified eagle’s medium
*E. coli*	*Escherichia coli*
ELISA	Enzyme-linked immunosorbent assay
H&E	Hematoxylin and eosin
IBDV	Infectious bursal disease virus
IPTG	Isopropyl-β-D-galactoside
SDS-PAGE	Sodium dodecyl sulphate polyacrylamide gel electrophoresis
SPF	Specific-pathogen-free
TEM	Transmission electron microscopy
VLPs	Virus-like particles
W.B	Western blotting
