# Supplementary material for: Recombinant B87 and HLJ0504 VP2 Vaccines Protect Chickens Against Infectious Bursal Disease
Source: Vet Sci. 2026 Sep 29;13(10):1033. doi: 10.3390/vetsci13101033 (PMC13709485; doi:10.3390/vetsci13101033)
Supplement: Supplementary file 1 [file vetsci-13-01033-s001.zip › Table S1.pdf]

**Table S1.** Experimental design for chicken immunization.

| Type | Group | Composition | Immunizing dose | Administration | Number of chickens | Age (days) at vaccination |
|---|---|---|---|---|---|---|
| Unpurified groups (VP2) | A | B87 VP2 (AGP 1:10) | 250 μL | Intramuscular | 10 | 28 |
| | B | B87 VP2 (AGP 1:20) | | | 10 | |
| | C | B87 VP2 (AGP 1:40) | | | 10 | |
| | D | 0504 VP2 (AGP 1:10) | | | 10 | |
| | E | 0504 VP2 (AGP 1:20) | | | 10 | |
| | F | 0504 VP2 (AGP 1:40) | | | 10 | |
| Purified groups (VLPs) | G | B87 VLPs (1 μg) | 250 μL | Intramuscular | 10 | 28 |
| | H | B87 VLPs (5 μg) | | | 10 | |
| | I | B87 VLPs (10 μg) | | | 10 | |
| | G | 0504 VLPs (1 μg) | | | 10 | |
| | K | 0504 VLPs (5 μg) | | | 10 | |
| | L | 0504 VLPs (10 μg) | | | 10 | |
| Control groups | M | Subunit vaccine a (AGP ≥ 16) | 300 μL | Intramuscular | 10 | 28 |
| | N | Inactivated vaccine | 300 μL | Intramuscular | 10 | |
| | O | Attenuated vaccine (B87 strain) | 1.5 feathers/1ml | oral | 10 | |
| | P | challenged control | 200 μL | oral | 10 | |
| | Q | PBS | 250 μL | Intramuscular | 10 | |
| | R | - | - | - | 5 | |
